# Detection of Genomic Structural Variants from Next-Generation Sequencing Data

**DOI:** 10.3389/fbioe.2015.00092

**Published:** 2015-06-25

**Authors:** Lorenzo Tattini, Romina D’Aurizio, Alberto Magi

**Affiliations:** ^1^Department of Neurosciences, Psychology, Pharmacology and Child Health, University of Florence, Florence, Italy; ^2^Laboratory of Integrative Systems Medicine (LISM), Institute of Informatics and Telematics and Institute of Clinical Physiology, National Research Council, Pisa, Italy; ^3^Department of Clinical and Experimental Medicine, University of Florence, Florence, Italy

**Keywords:** next generation sequencing, structural variants, copy number variants, statistical methods, whole-exome sequencing, whole-genome sequencing, amplicon sequencing

## Abstract

Structural variants are genomic rearrangements larger than 50 bp accounting for around 1% of the variation among human genomes. They impact on phenotypic diversity and play a role in various diseases including neurological/neurocognitive disorders and cancer development and progression. Dissecting structural variants from next-generation sequencing data presents several challenges and a number of approaches have been proposed in the literature. In this mini review, we describe and summarize the latest tools – and their underlying algorithms – designed for the analysis of whole-genome sequencing, whole-exome sequencing, custom captures, and amplicon sequencing data, pointing out the major advantages/drawbacks. We also report a summary of the most recent applications of third-generation sequencing platforms. This assessment provides a guided indication – with particular emphasis on human genetics and copy number variants – for researchers involved in the investigation of these genomic events.

## Introduction

Structural variants (SVs) are genomic rearrangements affecting more then 50 bp. The average SV size detected by the 1000 Genomes Project is 8 kbp (1000 Genomes Project Consortium et al., 2010), whereas a study based on tiling CGH array (Conrad et al., 2010) reports a four times larger value. SVs comprise balanced as well as unbalanced events, namely, variants altering the total number of base pairs in a genome. Thus, SVs include deletions, insertions, inversions, mobile-element transpositions, translocations, tandem repeats, and copy number variants (CNVs).

Several databases – e.g., the Database of Genomic Variants archive which reports structural variation identified in healthy control samples (DGVa^1^ ) – have been created for the collection of SVs data (Lappalainen et al., 2013). Public data resources have been developed with the purpose of supporting the interpretation of clinically relevant variants, e.g., dbVar^2^ , or collecting known disease genes (OMIM^3^ ) hit by SVs.

Structural variants account for 1.2% of the variation among human genomes while single nucleotide polymorphisms (SNPs) represent 0.1% (Pang et al., 2010). Notably, unbalanced events provide 99.8% of the entries reported in dbVar (Lin et al., 2014). CNVs may result in benign polymorphic variations or clinical phenotypes due to gene dosage alteration or gene disruption (Zhang et al., 2009). Though the impact of SVs in human genomics was first recognized by their presence in healthy individuals (Zhao et al., 2013), two models account for their association to human disease. Rare large events (<1%, hundreds kbp) have been related to neurological and neurocognitive disorders (Sebat et al., 2007; Girirajan et al., 2013), whereas multicopy gene families, which are commonly copy number variable, contribute to disease susceptibility.

Next-generation sequencing technologies (NGS) have been revolutionizing genome research [for a survey of NGS tools from quality check to variant annotation and visualization, see Pabinger et al. (2014)] as well as the study of CNVs (Duan et al., 2013; Zhao et al., 2013; Samarakoon et al., 2014; Tan et al., 2014; Alkodsi et al., 2015; Kadalayil et al., 2015) and SVs on the whole (Alkan et al., 2011a), replacing microarrays as the leading platform for the investigation of genomic rearrangement (Pinkel et al., 1998; Snijders et al., 2001; Iafrate et al., 2004; Sebat et al., 2007). NGS platforms are based on various implementations of cyclic-array sequencing (Shendure and Ji, 2008; Shendure et al., 2011). They allow for the sequencing of millions of short (few hundreds bp) DNA fragments (reads) simultaneously and may process a whole human genome in three days at 500-fold less cost than previous methods (Voelkerding et al., 2009; Metzker, 2010).

The 1000 Genomes Project applied methods based on all of the four approaches available for the detection of SVs, reporting false discovery rates ranging from 10 to 89%, remarkable differences in terms of genomic regions discovered, size range, and breakpoint precision (Mills et al., 2011; Teo et al., 2012).

## Overview of the Approaches

**Figure 1:**
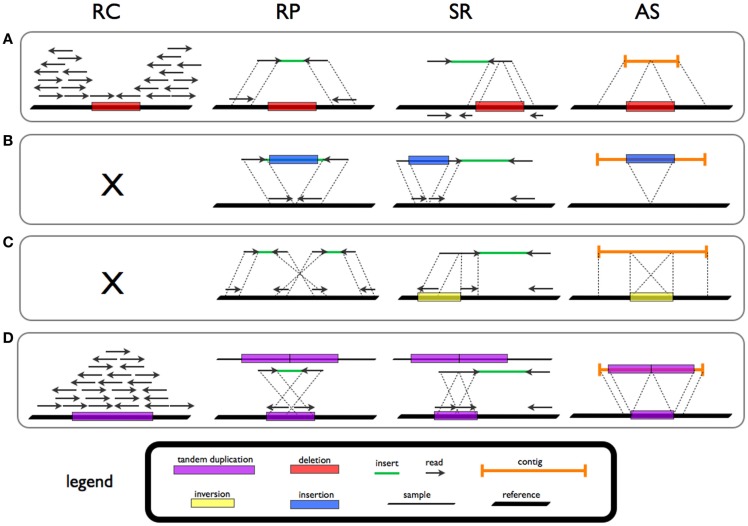
**Signatures and patterns of SVs for deletion (A), novel sequence insertion (B), inversion (C), and tandem duplication (D) in read count (RC), read-pair (RP), split-read (SR), and *de novo* assembly (AS) methods**.

Four strategies for the detection of SV signatures that are diagnostic of different rearrangements have been reported in the literature (Figure 1; Table 1).

**Table 1:** **A non-exhaustive summary of the tools/algorithms for the investigation of SVs, their input data (WGS, whole-genome sequencing; WES, whole-exome sequencing; CC, custom capture; AMS, amplicon sequencing), and their underling approach**.

Tool/algorithm	Input data	Method	Reference
EXCAVATOR	WES	RC	Magi et al. (2013)
ExomeCNV	WES	RC	Sathirapongsasuti et al. (2011)
CoNIFER	WES	RC	Krumm et al. (2012)
CODEX	WES	RC	Jiang et al. (2015)
XHMM	WES	RC	Fromer et al. (2012)
–	WES/CC	RC	Bansal et al. (2014)
ONCOCNV	AMS	RC	Boeva et al. (2014)
CNVnator	WGS	RC	Abyzov et al. (2011)
SegSeq	WGS	RC	Chiang et al. (2009)
CNAnorm	WGS	RC	Gusnanto et al. (2012)
CNAseg	WGS	RC	Ivakhno et al. (2010)
rSW-seq	WGS	RC	Kim et al. (2010)
cn.MOPS	WGS	RC	Klambauer et al. (2012)
JointSLM	WGS	RC	Magi et al. (2011)
ReadDepth	WGS	RC	Miller et al. (2011)
BIC-seq	WGS	RC	Xi et al. (2011)
PSCC	WGS	RC	Li et al. (2014)
CNV-seq	WGS	RC	Xie and Tammi (2009)
CLEVER	WGS	RP	Marschall et al. (2012)
BreakDancer	WGS	RP	Chen et al. (2009)
VariationHunter	WGS	RP	Hormozdiari et al. (2011)
PEMer	WGS	RP	Korbel et al. (2009)
MoDIL	WGS	RP	Lee et al. (2009)
Gustaf	WGS	SR	Trappe et al. (2014)
Socrates	WGS	SR	Schröder et al. (2014)
Splitread	WGS/WES	SR	Karakoc et al. (2012)
Cortex	WGS	AS	Iqbal et al. (2012)
Magnolya	WGS	AS	Nijkamp et al. (2012)
Tea	WGS	DC	Lee et al. (2012)
RetroSeq	WGS	DC	Keane et al. (2013)
Tangram	WGS	DC	Wu et al. (2014)
Mobster	WGS/WES	DC	Keane et al. (2013)

SVDetect	WGS	RC + RP	Zeitouni et al. (2010)
GASVpro	WGS	RC + RP	Sindi et al. (2012)
CNVer	WGS	RC + RP	Medvedev et al. (2010)
inGAP-sv	WGS	RC + RP	Qi and Zhao (2011)
Pindel	WGS	RP + SR	Ye et al. (2009)
LUMPY	WGS	RP + SR	Layer et al. (2014)
DELLY	WGS	RP + SR	Rausch et al. (2012)
PRISM	WGS	RP + SR	Jiang et al. (2012)
MATE-CLEVER	WGS	RP + SR	Marschall et al. (2013)
NovelSeq	WGS	RP + AS	Hajirasouliha et al. (2010)
HYDRA	WGS	RP + AS	Quinlan et al. (2010)
CREST	WGS	SR + AS	Wang et al. (2011)
SVseq	WGS	RC + SR	Zhang and Wu (2011)
SoftSearch	WGS/WES/CC	RP + SR	Hart et al. (2013)
Genome STRiP	WGS	RP + SR + RC	Handsaker et al. (2011)

*Methods designed using WGS data can, in principle, be used with WES data, though with limitations due to the intrinsic sparseness of WES data*.

Read-pair (RP) methods are based on the evaluation of the span and orientation of paired-end reads. Discordant pairs in which the mapping span and/or orientation of the read pairs are inconsistent with the expected insert size are collected. Several classes of SVs can be investigated by means of this approach. Read pairs mapping too far apart are associated to deletions while those found closer than expected are indicative of insertions. Furthermore, orientation inconsistencies can represent inversions and a specific class of tandem duplications.

Read-depth (or read count, RC) approaches assume a random (Poisson or modified Poisson) distribution in mapping depth and investigate the divergence from this distribution to highlight duplications and deletions (Magi et al., 2012). Sequencing of duplicated/amplified regions results in higher read depth while deleted regions show reduced read depth when compared to normal (e.g., diploid) regions.

Split-read (SR) methods allow for the detection of SVs with single base-pair resolution. The presence of a SV breakpoint is investigated on the basis of a split sequence-read signature breaking the alignment to the reference. A gap in the read is a marker of a deletion while stretches in the reference reflect insertions.

Theoretically, all forms of structural variation could be investigated by means of *de novo* assembly (AS) methods. *De novo* assembly refers to merging and ordering short fragments to reassemble the original sequence from which the short fragments were sampled (Earl et al., 2011). NGS data intrinsic characteristics, such as (short) read length, limit the use of AS approaches for variant investigation.

Moreover, a specific class of SV, mobile elements (ME) insertions, can be investigated exploiting discordant and clipped (DC) read information.

## Read Count Methods

Read count is suitable for the investigation of CNVs. RC methods comprise four steps: RC data preparation, data normalization, SV regions identification, and copy number estimation. Reads mapping to windows/bins of fixed size are counted (Yoon et al., 2009; Magi et al., 2011) and the results are normalized for the mitigation of local GC content and mappability effects.

The correlation between local GC content and read coverage has been detected through the analysis of data from several platforms (Harismendy et al., 2009). Mappability bias is due to repetitive regions within the human genome (Miller et al., 2011).

A segmentation step is necessary to split RC signal into segments characterized by a constant DNA copy number. Algorithms conceived for aCGH data such as the circular binary segmentation (CBS) algorithm (Campbell et al., 2008; Miller et al., 2011) and those based on hidden Markov models (HMM) (Magi et al., 2010) are used with this scope.

Copy number estimation can be tackled by means of two strategies. Both assume that the sequencing process is uniform. Thus, the number of reads mapping to a genomic region is expected to be proportional to the number of times the regions appears in the DNA sample. Three methods (Campbell et al., 2008; Yoon et al., 2009; Magi et al., 2011) estimate DNA copy number of all the detected regions rounding the median RCs (normalized to copy number 2) to the nearest integer, while CNVnator (Abyzov et al., 2011) uses RC signal normalized to the genomic average for the regions of the same length.

A considerable number of methods for the detection of CNV in whole-genome sequencing (WGS) data have been reported in the literature, including CNVnator, CNAnorm, CNAseg, rSW-seq, cn.MOPS, JointSLM, ReadDepth, and BIC-seq (Ivakhno et al., 2010; Kim et al., 2010; Abyzov et al., 2011; Magi et al., 2011; Miller et al., 2011; Xi et al., 2011; Gusnanto et al., 2012; Klambauer et al., 2012). Recently, PSCC (Li et al., 2014) has been compared with SegSeq (Chiang et al., 2009) and ReadDepth (Miller et al., 2011).

### CNV detection from whole-exome data

Due to the costs associated to WGS, the investigation of CNVs using whole-exome sequencing (WES) data is definitely an attractive perspective. Nevertheless, the sparse nature of the target and the non-uniform read-depth among captured regions make CNV detection from WES data awkward with respect to WGS [in particular, regarding the segmentation step as reported in Magi et al. (2013)].

Several tools have been reported in the literature for this purpose including ExomeCNV (Sathirapongsasuti et al., 2011), CoNIFER (Krumm et al., 2012), CNV-seq (Xie and Tammi, 2009), XHMM (Fromer et al., 2012), and recently EXCAVATOR (Magi et al., 2013) and CODEX (Jiang et al., 2015). Notably, the method developed by Bansal and co-workers (Bansal et al., 2014) allows for the analysis of NGS data generated from small subsets of the exome, namely custom capture (CC) data.

### Amplicon sequencing data

Amplicon sequencing (AMS) techniques have been reported in the literature in particular for clinical applications (Desai and Jere, 2012; Beadling et al., 2013).

Amplicon sequencing data show different biases in respect of WES data (Boeva et al., 2014). Data normalization can be less effective due to the limited number of target regions. Furthermore, protocols involved in the preparation of amplicon libraries result in high depth of coverage at the expense of coverage homogeneity.

The first method designed for the investigation of CNV from AMS data is ONCOCNV. Duplicate sequences are not removed, while RC is performed assigning “each read to only one amplicon region, the one with which the read alignment has the maximum overlap” (Boeva et al., 2014).

Data are then normalized with respect to library size assuming a similar efficiency of PCR amplification for all the targeted regions. GC content and amplicon length biases are corrected by means of a local polynomial regression fitting. Principal component analysis (PCA) is employed to construct a baseline reflecting the technological bias in control samples. The baseline is calculated by means of the first three principal components (calculated from control samples data). In order to define a significant threshold to call a copy number change, the standard deviation of the normalized RCs for each amplicon region is calculated.

This procedure is applied to data from test samples keeping the residuals of the linear regression of normalized RCs over the baseline calculated for the control samples.

Segmentation of the resulting signal profile is performed with CBS method (Venkatraman and Olshen, 2007). A segmentation and clustering approach (SCA) is used to define the copy number state (neutral, gain, or loss) of the segmented regions.

## Read-pair algorithms

As already mentioned, RP methods, as well as SR approaches, are suitable for the detection of several classes of SV including insertions of novel sequences and inversions. Notably, RP algorithms cannot detect the signatures of novel sequence insertions larger than the average insert size. Several tools based on the detection of SV signatures from *clusters* of read-pairs have been reported in the literature including BreakDancer, VariationHunter, PEMer, and GASV (Chen et al., 2009; Hormozdiari et al., 2009, 2011; Korbel et al., 2009; Sindi et al., 2009). Remarkably, PEMer can be exploited for the identification of linked insertions (Medvedev et al., 2010).

Clusters can be defined according to two strategies. The standard clustering strategy relies on two parameters: the minimum number of pairs with similar signature and the maximum value of the mean insert size standard deviation for a pair to be considered concordant. The maximum standard deviation value is fixed and events spanning the same locus, resulting in a small value of the insert size standard deviation, may be missed.

Distribution-based approaches, e.g., MoDIL (Lee et al., 2009), exploit the local distribution of all the mappings spanning a particular location on the genome. A read cluster is generated when the local distribution is shifted in respect to the typical insert size distribution. This approach allows for the detection of smaller events (e.g., compared with VariationHunter). The presence of two superimposed insert size distributions can be also detected, thus allowing for the discrimination of homozygous and heterozygous variants.

In the first implementations of the approach, e.g., BreakDancer (Chen et al., 2009), reads with multiple mappings were discarded. Thus, repetitive regions of the genome (including segmental duplications and copy-number amplifications) could not be investigated. Notably, BreakDancer allows for the identification of inter- and intra-chromosomal translocations. Tools such as MoDIL and VariationHunter or, more recently, CLEVER (Marschall et al., 2012) deal with multiple mapping reads [aligned, for instance, with mrFast (Alkan et al., 2009), Mosaik (Lee et al., 2014), BWA (Li and Durbin, 2010), or Bowtie (Langmead et al., 2009)]. CLEVER uses an insert size-based approach to build a graph with all reads and evaluates SV from maximal cliques. It is particularly well-tuned for the investigation of insertions and deletions of 50–100 bp.

## Split-read approaches

Though SR methods were conceived for Sanger sequencing reads (Mills et al., 2006), algorithms such as Pindel, Splitread, and Gustaf (Ye et al., 2009; Karakoc et al., 2012; Trappe et al., 2014) use paired-end NGS reads to identify SVs (or indel) events. SR approaches take advantage of one-end anchored reads, namely those pairs in which “one end is anchored to the reference genome and the other end maps imprecisely owing to the presence of an underlying structural variant or indel breakpoint” (Karakoc et al., 2012). SR-based tools can be applied solely to unique reference regions.

Pindel uses pattern growth for optimal matching in target regions, exploiting reads mapped with SSAHA2 [Sequence Search and Alignment by Hashing Algorithm, Ning et al. (2001)], BWA, or Mosaik. It must be stressed that the latest version of Pindel integrates RP to the SR information (Lin et al., 2014). Splitread searches for clusters of split reads using balanced splits as seeds. Splitread can detect, at least in theory, deletions without size limitation, while for insertions the size spectrum depends on the sequencing library. Insertions shorter than the read length can be accurately identified but larger insertions can only be approximately characterized within the insert size (Karakoc et al., 2012). Splitread is suitable for WGS/WES reads aligned using mrsFAST (Hach et al., 2010) to discover indels, SVs, *de novo* events, and pseudogenes.

Recently, Socrates (a SR method designed for cancer genomics) was compared to several tools (Schröder et al., 2014), including BreakDancer, CLEVER, CREST (Wang et al., 2011), DELLY (Rausch et al., 2012), Pindel, and PRISM (Jiang et al., 2012).

## Assembly based tools

*De novo* assembly allows – at least in principle – for the detection of all the forms of structural variation but the application of this approach is still challenging due to the limited length of NGS reads (Alkan et al., 2011a; O’Rawe et al., 2015).

AS methods were first exploited for Sanger sequencing data (characterized by read length between 300 and 1000 bp). The original *string graph approach* has been extended to de Bruijn graphs. The Assemblathon competition (Earl et al., 2011) produced a detailed comparison among *de novo* assemblers, including Phusion2 (Mullikin and Ning, 2003), SGA (Simpson and Durbin, 2010, 2012), Quake (Kelley et al., 2010), the first implementation of SOAPdenovo (Li et al., 2010; Luo et al., 2012), and ALLPATHS-LG (Gnerre et al., 2011), based on simulated data.

Two AS based callers have been reported in the literature for the investigation of SVs. Magnolya (Nijkamp et al., 2012) uses a Poisson mixture model (PMM) for CNV detection from contigs co-assembled from NGS sequencing data. The authors use an overlap-layout-consensus assembler to generate a contig string graph. Contig string graphs are characterized by nodes representing reads and edges representing an overlap. The final form of the graph is produced by transitive reduction – which removes redundant edges – and by unitigging (i.e., collapsing simple paths without branches) (Myers, 2005). In the resulting contig string graph, each node represents a collapsed set of reads called *contig*. Finally, the PMM approach for modeling read count is introduced to estimate the copy number of a contig. Once the model has been corrected for the presence of repetitive regions in the genome and prior knowledge on ploidy has been included, the model with the optimal number of Poisson distributions is selected by means of the lowest Bayesian information criterion. Integer copy numbers can be thus inferred by maximum *a posteriori* estimation. Remarkably, the method can be applied when no reference is available but – as already stressed – it is limited by the short read length typical of NGS platforms.

Cortex uses colored de Bruijn graphs with colors of both edges and nodes representing different samples and, possibly, reference sequences or known variants to assemble NGS reads. “The graph consists of a set of nodes representing words of length *k* (*k*-mers). Directed edges join *k*-mers seen consecutively in the input” (Iqbal et al., 2012). The package includes four algorithms for variant discovery. For example, the *bubble calling* algorithm may be exploited for the detection of variant bubbles in a colored de Bruijn graph from a single diploid individual. It must be stressed that using a reference genome aids the identification of variants while it is indispensable for the investigation of homozygous variant sites. Nevertheless, the sensitivity of the method decreases with the size of the variant. The tool has been extensively tested on human data.

## Combined Methods

None of the aforementioned approaches is capable of capturing the full spectrum of SV events with high sensitivity and specificity. RC methods can accurately predict absolute copy numbers but the breakpoint resolution is often inadequate and events such as inversions and novel sequence insertions cannot be detected. On the other hand, RP and SR approaches show low sensitivity in repetitive regions. Several packages combining different approaches for the investigation of SVs have been reported.

Combining RC for the detection of large events and RP for accurate identification of breakpoints can reduce the number of false positive calls [SVDetect (Zeitouni et al., 2010), CNVer (Medvedev et al., 2010), GASVPro (Sindi et al., 2012), and inGAP-sv (Qi and Zhao, 2011)]. Genome STRiP (Handsaker et al., 2011) exploits RP, RC, SR, and population-scale patterns to detect genome structural polymorphisms.

Packages implementing RP and (local) AS have been also reported [NovelSeq (Hajirasouliha et al., 2010), HYDRA (Quinlan et al., 2010)] as well as tools exploiting SR and RC/RP such as SVseq, MATE-CLEVER, and PRISM (Zhang and Wu, 2011; Jiang et al., 2012; Marschall et al., 2013). PRISM was tested on simulated data and compared with Pindel, SVseq, Splitread, and CREST. Notably, DELLY is suitable for detecting copy-number variable deletion and tandem duplication events as well as balanced rearrangements such as inversions or reciprocal translocations (Rausch et al., 2012), while SoftSearch (Hart et al., 2013) is designed for WGS, WES, and CC data. Recently, LUMPY has been shown to be “especially pronounced when evidence is scarce, either due to low coverage data or low variant allele frequency” (Layer et al., 2014). LUMPY is designed to integrate signals rather then refining primary signal with a secondary one. Furthermore, the tool combines different types of evidence from multiple samples.

## Detection of Mobile Elements

Mobile elements are repetitive DNA sequences that can change position within the genome (Lander et al., 2001). Due to this intrinsic characteristic, their detection is challenging. Latest estimates suggest that more than half of the human genome is repetitive or repeat-derived (de Koning et al., 2011). Though the DC approach can be ascribed to RP and SR methods, “the mates of the anchoring reads are then mapped to a custom but configurable library of known active ME consensus sequences” (Thung et al., 2014).

Among WGS tools, Tangram (Wu et al., 2014), a tool developed using Mosaik (Lee et al., 2014) alignments (though it may use alignments produced by other mappers), Next-Generation VariationHunter (Hormozdiari et al., 2010), Tea (Lee et al., 2012), RetroSeq eKeane:2013kq, and Mobster (Thung et al., 2014) have been reported in the literature.

## Conclusion

Overall, all the approaches discussed are fairly limited with respect to repeated regions of the reference genome (Alkan et al., 2009, 2011b). The complete range of structural DNA variation cannot be investigated with a single tool (Mills et al., 2011), though combined methods may aid the discovery of SV. Three pipelines integrating different tools exploiting WGS data have been reported in the literature (Wong et al., 2010; Lam et al., 2012; Mimori et al., 2013). WES data can be exploited for the investigation of SVs by means of RC, SR, and RP methods – though with limitations due to the intrinsic sparseness of exomic data.

Each method for the detection of SVs shows advantages/drawback. RC methods are particularly well-suited for the investigation of a particular class of SV, namely CNV. Notably, RC can be used to predict absolute copy number. A major drawback of RC tools is the poor breakpoint resolution. Furthermore, they cannot distinguish tandem from interspersed duplications. SR algorithms can accurately predict SV breakpoint (down to single-base resolution) as well as AS methods. Finally, the RP and SR approaches can be applied for the investigation of the widest range of SV classes (i.e., deletions, inversions, novel sequence insertions, tandem duplications), though both cannot be exploited for the calculation of absolute copy number.

The advent of third-generation sequencing (TGS) technology may contribute to overcome these issues (Schadt et al., 2010; Niedringhaus et al., 2011; Pareek et al., 2011; Venkatesan and Bashir, 2011). TGS single-end reads, characterized by read length up to thousands base pairs, may boost AS methods and the application of mapping algorithms allowing for split alignment such as BWA (Li and Durbin, 2010), LAST (Kiełbasa et al., 2011) and BLASR (Chaisson and Tesler, 2012). Though TGS platforms rely on different chemistry, reads produced by platforms, such as PacBio RS (Kim et al., 2014) and Oxford Nanopore MinION (Bayley, 2015), show similar read length and base-calling accuracy (~85%) (Quail et al., 2012; Quick et al., 2014; Ashton et al., 2015; Chaisson et al., 2015). Recent works have demonstrated that these technologies allow for the investigation of complex repetitive regions of the human genome (Chaisson et al., 2015) as well as the structure of complex antibiotic resistance islands in *Salmonella typhi* (Ashton et al., 2015) and tandem repeats in human bacterial artificial chromosome (Jain et al., 2015).

## Conflict of Interest Statement

The authors declare that the research was conducted in the absence of any commercial or financial relationships that could be construed as a potential conflict of interest.

## Footnotes

^1^http://www.ebi.ac.uk/dgva

^2^http://www.ncbi.nlm.nih.gov/dbvar

^3^http://www.omim.org
